# Socio-demographic characteristics influencing cervical cancer screening intention of HIV-positive women in the central region of Ghana

**DOI:** 10.1186/s40661-018-0060-6

**Published:** 2018-03-07

**Authors:** Nancy Innocentia Ebu

**Affiliations:** Department of Public Health, School of Nursing and Midwifery, University of Cape Coast, Cape Coast, Ghana

**Keywords:** Cervical cancer screening, Cervical cancer, Socio-demographics, HIV-positive women, Intention, Ghana, Developing countries

## Abstract

**Background:**

The burden of HIV and cervical cancer is concentrated in sub-Saharan Africa. Women with HIV are more likely to have persistent HPV infection leading to cervical abnormalities and cancer. Cervical cancer screening seems to be the single most critical intervention in any efforts to prevent cervical cancer. The purpose of this study was to determine the socio-demographic factors influencing intention to seek cervical cancer screening by HIV-positive women in the Central Region of Ghana.

**Methods:**

A descriptive cross-sectional study involving a convenience sample of 660 HIV-positive women aged 20 to 65 years receiving antiretroviral therapy in HIV care centres in the Central Region of Ghana was conducted using an interviewer-administered questionnaire. The data were summarised and analysed using frequencies, percentages and binary logistic regression.

**Results:**

The study revealed that 82.0% of HIV-positive women intended to obtain cervical cancer screening. Level of education was a determinant of cervical cancer screening intention. HIV-positive women with low levels of education were 2.67 times (95% CI, 1.61–4.42) more likely to have intention to screen than those with no formal education. Those with high levels of education were 3.16 times (95% CI, 1.42–7.02) more likely to have intention to screen than those with no formal education. However, age, religion, marital status, employment status, and ability to afford the cost of cervical cancer screening were not determinants of intention to screen.

**Conclusions:**

Education of women of all ages needs to be a priority, as it could enable them to adopt appropriate health behaviours and engage in cervical cancer screening. Additionally, interventions to improve understanding of cervical cancer screening among HIV-positive women are highly recommended. These include health education about the disease and availability of screening options in HIV/AIDS care centres.

## Background

Cancer of the cervix is an important concern in global health. Globally, cervical cancer is the second most prevalent cancer that impacts the health and mortality of women. It is estimated that 500,000 cases are diagnosed yearly, with over 250,000 deaths as a result of the condition [1]. Cervical cancer is a major cause of morbidity and mortality among women in poor resource settings, especially in Africa [1]. Significant proportions of cancers (over 80%) that occur in sub-Saharan Africa are discovered in late stages, mostly due to lack of information and limited access to preventive services [2, 3]. Late-stage disease is associated with low survival rates after surgery or radiotherapy. In addition, treatment options may not be available in developing economies, or they may be too costly and inaccessible to vulnerable women. Cervical cancer is potentially preventable, as effective screening programmes can lead to a significant reduction in the morbidity and mortality associated with this cancer [4]. The Global Health Group reports that more than 288,000 women die each year worldwide due to cervical cancer and the disease mostly affects vulnerable and disadvantaged women in society [5]. Most screening activities in developing countries do not reach vulnerable women, and consequently a high proportion of cervical cancer cases are diagnosed at an advanced stage [6].

The burden of HIV and cervical cancer is concentrated in sub-Saharan Africa. Women with HIV are more likely to have persistent HPV infection leading to cervical abnormalities and cancer [7, 8]. A systematic review reports that HIV infection could lead to a faster progression of cervical cancer due to a strong association between HIV infection and invasive cervical cancer [9]. This suggests that HIV-positive women need to screen early for an HIV-related cancer like cervical cancer to reduce risks of cancer. Previous studies conducted in sub-Saharan cultures have posited that socio-demographics, such as income, age and religion [10–15], level of education, marital status and age [16–18], influenced cervical cancer screening. However, the Health Belief Model (HBM) suggests that socio-demographic factors are modifiable variables which may influence an individual to engage in health behaviour [19]. Additionally, in Ghana, although cervical cancer may impact the health of HIV-positive women, few of the empirical works on cervical cancer screening focused on the attitudes and knowledge of women in general [20–22], and did not include HIV-positive women who are the most vulnerable group. This study therefore hypothesised that socio-demographic factors predict intention of HIV-positive women to seek cervical cancer screening.

## Methods

A descriptive cross-sectional study was conducted in the Central Region of Ghana. The region is located in the southern part of Ghana. The region recorded an HIV prevalence of 1.8 in the 2016 HIV sentinel survey, which suggests the presence of HIV infection in the region [23]. The study population, comprised all HIV-positive women in the Central Region, was estimated to be 6019 [24]. However, the accessible population was HIV-positive women receiving care in the various antiretroviral care centres in the Central Region, and was estimated to be 3483 [24]. The study includes HIV-positive women who were between the ages of 20 to 65 years, and had been receiving regular care in health facilities that provide care to people living with HIV/AIDS (PLWHIV) in the Central Region.

However, terminally ill clients and those with dementia were excluded as they might not be in a position to provide valid responses. Using a power formula derived by Ogah [25], a sample size of 660 HIV-positive women was estimated for the study. Six health facilities that provide care for PLWHIV in the Central Region were randomly selected for the study. The study also employed the probability proportionate to size sampling in determining the number of HIV-positive women to participate in the study from the facilities selected for the study. Accidental quota sampling technique was used in selecting HIV-positive women from the various health facilities until the sample size was reached.

### Data collection

A questionnaire was designed to identify the socio-demographic characteristics of HIV-positive women that influence intention to screen. It was guided by the HBM and Theory of Planned Behaviour. The questions were adopted from Ebu et al. [22] and Mupepi et al. [26]. The socio-demographic information considered included: age, marital status, religion, level of education, employment status and ability to afford the cost of cervical cancer screening. The independent variables for the study were; level of education, marital status, age, employment status, religion, and ability to afford the cost of cervical cancer screening. The dependent variable was intention to obtain cervical cancer screening. Data were collected from HIV-positive women who visited the health facilities during the period of data collection and volunteered to participate in the study. The data collectors were six HIV nurse prescribers (nurses who have been trained to provide antiretroviral therapy services to clients) who were trained for that purpose. The data was collected over a ten-week period. The Institutional Review Board of the University of Cape Coast and Ethical Review Committee of the Ghana Health Service gave ethical approval for the study to be conducted. Written informed consent was obtained from all participants.

### Data analysis

Data were analysed using frequencies, percentages and binary logistic regression analysis. Independent variables were categorised. Levels of education were high level of education (tertiary school), low level of education (primary and secondary), and no formal education. Marital status was married (married or cohabiting) or unmarried (single, divorced, or widowed). Employment status was working or non-working (student, retired, or unemployed). Religion comprised Christians and Muslims. Age was classified by 30 years and above and under 30. The ability to afford cervical cancer screening was classified as very affordable, fairly affordable or not affordable. The independent variables were categorical and measured on the nominal scale. The dependent variable was intention to obtain cervical cancer screening and was measured as either “Yes” or “No”.

## Results

The descriptive statistics of the respondents showed that 79.2% (*n* = 523) were above 30 years old, and 20.8% (*n* = 137) were under 30. With regard to their marital status, 53.2% (*n* = 351) were married while 46.8% (*n* = 309) were not married. A high proportion of the respondents, 89.7% (*n* = 592), were Christians while 10.3% (*n* = 68) were Muslims. Again, 21.7% (*n* = 143) were not formally educated, 62.1% (*n* = 410) had low level of education and only 16.2% (*n* = 107) had high level of education. Additionally, 54.2% (*n* = 358) were working while 45.8% (*n* = 302) were not working. More than half of the respondents, 55.3% (*n* = 365) viewed the cost of screening as not affordable while 44.7% (*n* = 295) perceived it as affordable. In connection with the intention of the respondents, 82.0% (*n* = 540) of HIV-positive women had intention to obtain cervical cancer screening while 18.0% (*n* = 120) did not have any intention. The results in Table 1 show that low levels of education (primary and secondary) and high education (tertiary) statistically significantly contributed to intention to screen, with *p*-values of 0.001 and 0.005 respectively using respondents with no formal education as the reference category. Respondents with a low level of education were 2 times more likely to have intention to screen [OR] 2.67 (95%CI = 1.61–4.42). Comparatively, those with tertiary or a high level of education were approximately 3 times more likely to have intention to screen [OR] 3.16 (95% CI = 1.42–7.02). However, marital status (*p* = 0.492, OR = 1.17, [95% CI, 0.74–1.86]), religion (*p* = 0.808, OR = 1.09, [95% CI, 0.55–2.18]), age (*p* = 0.704, OR = 1.12, [95% CI, 0.63–2.00]), employment status (*p* = 0.045, OR = 1.63, [95% CI, 1.01–2.64]), and ability to afford the cost of cervical cancer screening (*p* = 0.411, OR = 1.25, [95% CI, 0.73–2.13]) did not contribute statistically significantly to the prediction of intention in the model.

**Table 1:** Binary Logistic Regression to Predict Intention to Seek Cervical Cancer Screening based on Socio-Demographic Characteristics

Variables	n (%)	*β*	Wald	*p-*value	OR	95% CI for OR	
						Lower	Upper
Level of Education							
High	93 (17)	1.15	7.97	0.005	3.16	1.42	7.02
Low	350 (65)	0.98	14.35	0.001	2.67	1.61	4.42
No education (Ref.)	97 (18)						
Marital Status							
Married	297 (55)	0.16	0.47	0.492	1.17	0.74	1.86
Not married (Ref.)	243 (45)						
Religion							
Christianity	486 (90)	0.09	0.06	0.808	1.09	0.55	2.18
Islam (Ref.)	54 (10)						
Age (in years)							
30 and above	427 (79)	0.11	0.15	0.704	1.12	0.63	2.00
Under 30 (Ref.)	113 (21)						
Employment Status							
Working	311 (58)	0.49	4.02	0.045	1.63	1.01	2.64
Not working (Ref.)	229 (42)						
Ability to Afford Cervical Cancer Screening							
Affordable	255 (47)	0.22	0.68	0.411	1.25	0.73	2.13
Not affordable (Ref.)	285 (53)						
Constant		2.16	35.35	0.001	8.70		

## Discussion

The findings suggest that respondents with low to high levels of education are likely to screen than those with no formal education. A possible explanation for this outcome is that women who are educated may have increased understanding of health care services. Education seems to be an enabling factor that can help women to have a better understanding about cervical cancer and the screening facilities available. Educated women may also be in a better position to evaluate their level of risk concerning the disease. The level of educational attainment could facilitate decisions in accessing health services including cervical screening. Several studies have affirmed education as an important predictor of intention to screen and cervical cancer screening behaviour [27–30].

The present finding is consistent with previous studies, which could be due to the similarity in how level of education was measured [16, 29]. Level of education is known to be a modifiable factor, as it is known to modify other constructs in the HBM. Education is an individual characteristic that can influence subjective perception of screening [19, 31]. It has commonly been assumed that women with some level of education have better chances of using maternal health care services compared to those without any formal education [12, 32]. Education has a high tendency of changing beliefs and unfavourable behaviours if interventions are designed and targeted to address specific notions in respect to health and illness [33].

The findings suggest that women with no formal education may not have intention to seek cervical screening. Women who are illiterate or have not had any form of education may have poor access to health services and experience low quality of life. Importantly, they may delay in seeking health care, even when symptoms of the disease are obvious, compared to better educated women, who may respond faster [33]. Other empirical works have demonstrated that illiterate women may be less likely to seek cervical cancer screening [32, 34, 35]. Despite the heterogeneity in the methodology and in the characteristics of the population employed in these studies, the findings suggest that women with no formal education may be at high risk of contracting cervical cancer. Education, therefore, allows women to have improved socio-economic status and decreased morbidity and mortality, as they become more empowered to control the determinants of their health [36].

Marital status was not a determinant of intention to screen. A possible explanation could be due to how this concept was measured in this study, as those who were cohabiting were regarded as being in marital relationship. Additionally, respondents who are divorced and widowed were added to single women and considered unmarried. This finding confirms previous studies in which no significant relationship was found between those who were married and the unmarried in relation to cervical cancer screening intention [17, 29, 32]. Although there was heterogeneity in the methodology employed in these studies, the results were similar to the present study because the studies focused on women and the conceptual basis could be linked to the HBM. Ogunwale et al. explained that HIV-positive women who had not had cervical cancer screening were younger and had multiple sexual partners or had had sexual intercourse with a man with multiple sexual partners [37]. It is well documented that in Ghana, and other African societies, men tend to dominate affairs of women at the household level, including in decisions about seeking preventive healthcare [38]. In Nigeria, marital status was found not to be a predictor of cervical cancer screening uptake, as women had abysmally low knowledge and poor perception of the disease [39].

Similarly, respondents’ religion did not statistically contribute significantly to the prediction of intention to screen. The result is consistent with that of Ezechi et al. [28] and Modibbo et al. [14], in which religion did not predict cervical cancer screening intention. Categorisation of respondents’ religion in these previous studies was similar to that of the present study, and that may have influenced the outcome. Previous studies explained that, in the African context, religious and cultural beliefs of modesty, cervical cancer being viewed as a curse from God and a shameful disease associated with immorality [14], and male dominance over decisions about seeking health care may create barriers for many women [38].

Age also was not a determinant of cervical cancer screening intention. A possible explanation for this outcome could be that age was dichotomised into those under 30 and above 30 in this study. It could also be related to the fact that 79% of HIV-positive women with intention to screen were aged 30 years and above. This finding is consistent with previous studies in which age did not significantly predict willingness to have Pap test [17, 32]. This suggests that age is not an essential factor that may facilitate HIV-positive women’s intention to seek screening. It seems regularly seeking preventive health services, specifically cervical cancer screening, is not well grounded in the culture of Ghanaians, which may suggest that women of all ages may not seek screening services. Perhaps in Ghana and some African cultures, utilisation of preventive health services and health-care-seeking behaviours are not well defined. Consequently, most women intend to seek or actually seek health care when symptoms of the disease are conspicuous [40].

Statistically, employment status did not contribute significantly to the prediction of intention to seek cervical cancer screening. A possible explanation for this outcome is that, although employment provides a sense of self-esteem and could result in personal fulfillment, HIV-positive women may have issues with stigma and entertain fears that other people may get to know of their HIV status. This could potentially deter those who work and could afford cervical cancer screening from engaging in such an important behaviour. In addition, some of these women may lack knowledge about cervical cancer and they may also have specific barriers regarding screening. In this study, 42% of HIV-positive women with intention to screen were not working. The fact that these women were not in any formal job could affect their perception of their intention to seek cervical cancer screening. The finding confirms previous studies in which employment status was not a determinant of screening [41, 42]. It seems employment may not guarantee intention to participate in a health promotion activity. This suggests that other important factors could influence intention. HIV-positive women encounter unique problems, including stigma, discrimination, and fear of obtaining a positive cervical cancer screening result [43–46], despite being in active employment and having the financial resources for health. It could be explained that the socialisation processes of young and mature women in the Ghanaian culture’s strict traditions can deprive women of status, which can affect their pattern of health care seeking [47].

It is evident from the findings that ability to afford cervical cancer screening was not a determinant of intention to screen. A possible explanation for this outcome is that HIV-positive women have unique challenges regarding cervical cancer screening, as 53% of HIV-positive women with intention to screen perceived the cost as not being affordable. In previous studies conducted in developing settings, the ability to afford cervical cancer screening did not result in cervical cancer screening intention [28, 46, 48]. The ability to afford cervical cancer screening is a factor which has the potential of modifying perception constructs in the HBM [19]. However, HIV-positive women may have low socio-economic status and self-esteem that can impact utilisation of health services [49].

Previous studies found cost of cervical cancer screening to be a critical barrier to screening [50–53], if women perceived the cost to be high. One previous study reported that women may be willing to have cervical cancer screening if employers pay for the cost of screening [54]. This demonstrates an urgent need for women to be supported to act on their intention to screen [55]. For instance, in Ghana, although women play a critical role in nation building and form approximately 50% of the workforce, they are mostly involved in informal sector jobs such as trading and farming. Only 1% are in administrative positions in the public sector [56]. This suggests that it may be difficult for HIV-positive women to pay for cervical cancer screening which costs from 9.2 to 14.7 euros.

## Conclusions

The findings imply that some level of education encourages women to have positive attitudes and intention toward cervical cancer screening. Cervical cancer screening education should be designed to target HIV-positive women, especially those who are not literate since they may be at increased risk of developing the disease. It is critical that education of both young and mature women be promoted, as it may form an integral part in ensuring positive health behaviours. These findings indicate that women who are not educated may not utilise health services, as misconceptions and personal beliefs about it could be a barrier [15, 25]. Therefore, the family, community, civil society and government should view female education as a priority. Education of women has a positive impact on them, their family and the entire society. Interventions that could improve HIV-positive women’s understanding of cervical cancer screening, including health education about the disease and screening options in HIV/AIDS care centres, are highly recommended. Age, marital status, employment status, religion, and ability to afford cervical cancer screening, on the other hand, do not appear to influence intention to engage in cervical cancer screening. These factors may not be critical in designing interventions for HIV-positive women in the Central Region of Ghana.

## Abbreviations

HBM: Health Belief Model
HIV: Human Immunodeficiency Virus
TPB: Theory of Planned Behaviour
WHO: World Health Organisation
